# Supplementary material for: Higher Dietary Intake of Advanced Glycation End Products Is Associated with Faster Cognitive Decline in Community-Dwelling Older Adults
Source: Nutrients. 2022 Mar 31;14(7):1468. doi: 10.3390/nu14071468 (PMC9003315; doi:10.3390/nu14071468)
Supplement: Supplementary file 1 [file nutrients-14-01468-s001.zip › nutrients-1618883-supplementary.pdf]

Questionnaire S1. Dietary advanced glycation end products (AGEs) questionnaire.

1. BEEF or PORK

1.1. In the past week have you eaten beef or pork including burgers, meatballs, pork chops, roast beef, steak, ribs or any other kind of beef or pork?

[INTERVIEWER: if the subject mentions he/she ate bacon, hot dogs or sausages, or deli meat, ask if he/she ate any other type of beef or pork since we ask about these items later on]
NO—continue to the next food item in question 2 (poultry)
YES—ask about frequency, serving size and preparation method
1 = Yes 2 = No

1.2. How many times did you eat beef or pork in the past week?

1 = 1 per week 2 = 2–3 per week 3 = 4–6 per week 4 = Daily 5 = More than 1 per day 6 = Don't know

1.3. How much did you eat each time?

[INTERVIEWER: if the subject mentions he/she ate a different amount each time, ask him/her to specify the amount that was eaten most of the time]
1 = Less than 3 ounces 2 = Between 3–6 ounces 3 = More than 6 ounces 4 = Don't know

1.4. How was the beef or pork cooked?

[INTERVIEWER: if the subject mentions he/she ate beef or pork using different cooking methods, ask him/her to specify the cooking method that was used the most]
1 = Boiled/canned/stewed 2 = Fried in a pan 3 = Roasted 4 = Broiled/grilled 5 = Oven fried/deep fried 6 = Don't know

2. POULTRY

2.1. In the past week have you eaten poultry including chicken, turkey, duck, chicken breasts, chicken nuggets, drumsticks or wings?

NO—continue to the next food item in question 3 (fish)
YES—ask about frequency, serving size and preparation method
1 = Yes 2 = No

2.2. How many times did you eat poultry in the past week?

1 = 1 per week 2 = 2–3 per week 3 = 4–6 per week 4 = Daily 5 = More than 1 per day 6 = Don't know

2.3. How much did you eat each time?

[INTERVIEWER: if the subject mentions he/she ate a different amount each time, ask him/her to specify the amount that was eaten most of the time]
1 = Less than 3 ounces 2 = Between 3–6 ounces 3 = More than 6 ounces 4 = Don't know

2.4. How was the poultry cooked?

[INTERVIEWER: if the subject mentions he/she ate poultry using different cooking methods, ask him/her to specify the cooking method that was used the most]
1 = Boiled/canned/stewed 2 = Fried in a pan 3 = Roasted 4 = Broiled/grilled 5 = Oven fried/deep fried 6 = Don't know

3. FISH

3.1. In the past week have you eaten fish including salmon, tuna, trout, tilapia or any other type of fish or seafood?

[INTERVIEWER: Crustaceans, such as crab, shrimp, lobster, count for this question.]
NO—continue to the next food item in question 4 (cheese)
YES—ask about frequency, serving size and preparation method
1 = Yes 2 = No

3.2. How many times did you eat fish in the past week?

1 = 1 per week 2 = 2–3 per week 3 = 4–6 per week 4 = Daily 5 = More than 1 per day 6 = Don't know

3.3. How much did you eat each time?

[INTERVIEWER: if the subject mentions he/she ate a different amount each time, ask him/her to specify the amount that was eaten most of the time]
1 = Less than 3 ounces 2 = Between 3–6 ounces 3 = More than 6 ounces 4 = Don't know

3.4. How was the fish cooked?

[INTERVIEWER: if the subject mentions he/she ate fish using several cooking methods, ask him/her to specify the cooking method that was used the most. Uncooked/raw fish should be coded as smoked/water canned.]
1 = Smoked/water canned 2 = Boiled/oil canned 3 = Poached 4 = Broiled/grilled/baked 5 = Oven fried/deep fried 6 = Don't know

4. CHEESE

4.1. In the past week have you eaten any type of cheese including American cheese,cheddar, mozzarella, brie, swiss, feta, cottage cheese or a similiar type?

[INTERVIEWER: Cream cheese does NOT count as a type of cheese.]
NO—continue to the next food item in question 5 (fats added to food)
YES—ask about frequency, serving size and preparation method
1 = Yes 2 = No

4.2. How many times did you eat cheese in the past week?

1 = 1 per week 2 = 2–3 per week 3 = 4–6 per week 4 = Daily 5 = More than 1 per day 6 = Don't know

4.3. Each time you ate cheese, what was the amount?

[INTERVIEWER: if the subject mentions he/she ate a different amount each time, ask him/her to specify the amount that was eaten most of the time]
1 = Less than or 1 slice (for American cheese, cheddar, mozzarella or a similar cheese) @//Less than 1 oz(for brie, swiss, feta) @//Less than 0.5 cup (for cottage or any other soft cheese 2 = 1–2 slices (for American cheese, cheddar, mozzarella or a similar cheese) @//1–2 oz (for brie, swiss, feta) @//Between 0.5–1 cup (for cottage or any other soft cheese) 3 = 3 or more slices(for American cheese, cheddar, mozzarella or a similar cheese), @//3 oz or more (for brie, swiss, feta) @//More than 1 cup (for cottage or any other soft cheese) 4 = Don't know

4.4. Do you eat regular cheese or low fat cheese most of the time?

[INTERVIEWER: if cottage cheese is the type of cheese eaten the most, then low fat should ALWAYS be selected.]
1 = Low fat 2 = Regular 3 = Don't know

5. SPREADS AND OILS (added to food)

5.1. In the past week have you added any spreads or oils to your food such as: butter/margarine, cream cheese, cooking oil, peanut butter or mayonnaise?

[INTERVIEWER: The following foods count as oil or spread: sour cream, nut butters, meat pâtés.]

[INTERVIEWER: The following foods DO NOT count as oil or spread: salad dressing, hummus, ketchup, mustard, honey, jam.]
NO—continue to the next food item in question 6 (processed foods)
YES—continue to the following question
1 = Yes 2 = No

5.2. How many times did you use these spreads, or oils in the past week?

[INTERVIEWER: list again if necessary- butter/margarine, cream cheese, cooking oil, peanut butter or mayonnaise. Salad dressing does NOT count as a type of oil or spread.]
1 = 1 per week 2 = 2–3 per week 3 = 4–6 per week 4 = Daily 5 = More than 1 per day 6 = Don't know

5.3. How many tablespoons did you eat each time?

[INTERVIEWER: if the subject mentions he/she ate more than one of the foods listed above per day, ask him/her to think of how many tablespoons he ate on average each time, across all types of spreads and oils]
1 = Less than 1 tbsp 2 = Between 1 tbsp–2 tbsp 3 = 3 or more tbsp 4 = Don't know

6. PROCESSED FOODS

6.1. In the past week have you eaten any of the following processed foods: pizza, hot dogs or sausages, bacon, or any type of deli meat?

NO—continue to the end of the questionnaire
YES—continue to the following question
1 = Yes 2 = No

6.2. How many times did you eat processed foods in the past week?

1 = 1 per week 2 = 2–3 per week 3 = 4–6 per week 4 = Daily 5 = More than 1 per day 6 = Don't know

6.3. What was the size of the serving you ate each time?

[INTERVIEWER: if the subject mentions he/she ate a different amount each time, ask him/her to specify the amount that was eaten most of the time]
1 = Small (1 or less slices of pizza, 2 or less hot dogs, 1 or less slice of bacon, 5 or less slices of deli meat) 2 = Medium (2–3 slices of pizza, 3–4 hot dogs, 2 slices of bacon, 10 slices of deli meat) 3 = Large (more than 3 slices of pizza, more than 4 hot dogs, more than 2 slices of bacon, more than 10 slices of deli meat) 4 = Don't know

INDIVIDUAL CLASS AGEs MEASURES

Beef score = beef frequency × beef quantity × beef preparation method
Poultry score = poultry frequency × poultry quantity × poultry preparation method
Fish score = fish frequency × fish quantity × fish preparation method
Cheese score = cheese frequency × cheese quantity × cheese fat
Spreads score = spreads frequency × spreads quantity
Processed foods score = processed foods frequency × processed foods quantity
OVERAL DAILY AGEs SCORE = (beef score + poultry score + fish score + cheese score + spreads score + processed foods score)/7

Additional detail on the methods used to define cognitive impairment: Neuropsychological tests do not measure cognition uniformly across different levels of education. An algorithm rated impairment in 5 cognitive domains (i.e., orientation, attention, memory, language, perception) based on educationally adjusted cutoff scores on 11 cognitive tests [1,2]. Second, after reviewing all cognitive data, education, occupation, and ratings of sensory problems, motor problems, and the participant's level of effort, a neuropsychologist agreed or disagreed with the algorithmic rating of each cognitive domain. In the event of disagreement, the neuropsychologist supplied a new impairment rating.

The neuropsychological tests were used to guide clinical judgment to enhance uniformity of clinical decisions across examiners and over time and to reduce bias based on age, sex, or race, both the neuropsychologist's and clinician's decisions were based on clinical judgment [1,2].

Annual clinical diagnosis was based on the recommendations of the joint working groups of the National Institute of Aging and Alzheimer's Disease Association (NIA-AA). A diagnosis of Alzheimer's dementia required meaningful decline in cognitive function that impaired the ability to work or perform usual activities [2,3]. Participants who did not meet the accepted criteria for dementia by the clinician but were judged to have cognitive impairment by the neuropsychologist and absence of dementia in the opinion of the clinician were classified with Mild Cognitive Impairment (MCI). MCI was classified as amnestic if the memory domain was impaired and non-amnestic if memory was not impaired, as previously reported [4,5]. These MCI criteria have been related to intermediate levels of cognitive decline [4,6] and Alzheimer's disease pathology [7] compared to dementia and no cognitive impairment.

Table S1. Dietary AGEs intake and declining cognitive abilities adjusting for sociodemographic and cardiovascular risks and diseases.

Cognitive Ability Ou-tocme	Model Term	Estimate (S.E, *p* Value)
Global Cognition	Time	−0.082 (0.017, <0.001)
	Time × dietary AGEs intake	**−0.003 (0.001, 0.013)**
Semantic Memory	Time	−0.088 (0.021, <0.001)
	Time × dietary AGEs intake	<0.001 (0.001, 0.859)
Episodic Memory	Time	−0.092 (0.026, 0.003)
	Time × dietary AGEs intake	**−0.004 (0.002, 0.013)**
Working Memory	Time	0.046 (0.026, 0.081)
	Time × dietary AGEs intake	−0.001 (0.002, 0.383)
Perceptual Speed	Time	−0.117 (0.021, <0.001)
	Time × dietary AGEs intake	**−0.003 (0.001, 0.048)**
Visuospatial Abilities	Time	−0.026 (0.030, 0.390)
	Time × dietary AGEs intake	−0.002 (0.002, 0.412)

Each of models shows the results for the model terms of "Time" i.e., the annual rate of change in global cognition and the interaction of baseline dietary advanced glycation end products (AGEs) score with "Time" from a single linear mixed-effect model which also included additional terms for age, sex, education, race, body mass index (BMI), cardiovascular risk factors and diseases, and their interaction with time. S.E, standard error.

Table S2. Dietary AGEs intake of specific food groups and decline in global cognition.

Food Group	Model Term	Estimate (S.E, *p* Value)
Meat	Time	0.076 (0.011, <0.001)
	Time × dietary AGEs intake	−0.004 (0.003, 0.237)
Poultry	Time	−0.079 (0.011, <0.001)
	Time × dietary AGEs intake	−0.004 (0.004, 0.285)
Fish	Time	−0.077 (0.011, <0.001)
	Time × dietary AGEs intake	−0.007 (0.004, 0.084)
Cheese	Time	−0.076 (0.011, <0.001)
	Time × dietary AGEs intake	−0.005 (0.005, 0.374)
Spread	Time	−0.077 (0.011, <0.001)
	Time × dietary AGEs intake	−0.003 (0.006, 0.624)
Processed foods	Time	−0.077 (0.011, <0.001)
	Time × dietary AGEs intake	−0.003 (0.003, 0.352)

Each of models shows the results for the model terms of "Time" i.e., the annual rate of change in global cognition and the interaction of baseline dAGEs score of each of the food groups with "Time" from a single linear mixed-effect model which also included additional terms for age, sex, education, race, BMI, and their interaction with time.

Table S3. Spearman correlations among dietary AGE (dAGE) categories.

Correlation	Beef	Poultry	Fish	Cheese	Spreads
**Poultry**	0.17				
**Fish**	0.08	0.13			
**Cheese**	0.12	0.08	0.01		
**Spreads**	0.12	0.05	0.06	0.19	
**Processed foods**	0.1337	0.08	0.09	0.17	0.15

References

1. Bennett, D.A.; Wilson, R.S.; Schneider, J.A.; Evans, D.A.; Beckett, L.A.; Aggarwal, N.T.; Barnes, L.L.; Fox. J.H.; Bach, J. Natural history of mild cognitive impairment in older persons. *Neurology*. **2002**, *59*, 198–205.
2. Bennett, D.A.; Schneider, J.A.; Aggarwal, N.T.; Arvanitakis, Z.; Shah, R.C.; Kelly, J.F.; Fox, J.H.; Cochran, E.J.; Arends, D.; Treinkman, A.D; Wilson, R.S. Decision rules guiding the clinical diagnosis of Alzheimer's disease in two community-based cohort studies compared to standard practice in a clinic-based cohort study. *Neuroepidemiology*. **2006**, *27*, 169–176.
3. McKhann, G.; Drachman, D.; Folstein, M.; Katzman, R.; Price, D.; Stadlan, E.M. Clinical diagnosis of Alzheimer's disease: report of the NINCDS-ADRDA Work Group under the auspices of Department of Health and Human Services Task Force on Alzheimer's Disease. *Neurology*. **1984**, *34*, 939–944.
4. Wilson, R.S.; Leurgans, S.E.; Boyle, P.A.; Bennett, D.A. Cognitive decline in prodromal Alzheimer disease and mild cognitive impairment. *Arch Neurol*. **2011**, *68*, 351–356.
5. Wilson RS, Schneider JA, Arnold SE, Tang Y, Boyle PA, Bennett DA. Olfactory identification and incidence of mild cognitive impairment in older age. *Arch Gen Psychiatry*. **2007**, *64*, 802–808.
6. Boyle, P.A.; Wilson, R.S.; Aggarwal, N.T.; Tang, Y.; Bennett, D.A. Mild cognitive impairment: risk of Alzheimer disease and rate of cognitive decline. *Neurology*. **2006**, *67*, 441–445.
7. Schneider, J.A.; Arvanitakis, Z.; Leurgans, S.E.; Bennett, D.A. The neuropathology of probable Alzheimer disease and mild cognitive impairment. *Ann Neurol*. **2009**, *66*, 200–208.
